# Multidimensional Criteria for Virtual Screening of PqsR Inhibitors Based on Pharmacophore, Docking, and Molecular Dynamics

**DOI:** 10.3390/ijms25031869

**Published:** 2024-02-03

**Authors:** Haichuan Xiao, Jiahao Li, Dongdong Yang, Jiarui Du, Jie Li, Shuqi Lin, Haibo Zhou, Pinghua Sun, Jun Xu

**Affiliations:** 1 College of Pharmacy, Jinan University, Guangzhou 510632, China; xhc991288@stu2021.jnu.edu.cn (H.X.); ljhhhh@stu2021.jnu.edu.cn (J.L.); a970216469@stu2021.jnu.edu.cn (D.Y.); djr2003@stu2021.jnu.edu.cn (J.D.); 2160712726lj@stu2022.jnu.edu.cn (J.L.); kklinn@stu2021.jnu.edu.cn (S.L.); haibo.zhou@jnu.edu.cn (H.Z.); 2 State Key Laboratory of Bioactive Molecules and Druggability Assessment, Jinan University, Guangzhou 510632, China; 3 Key Laboratory of Xinjiang Phytomedicine Resource and Utilization, Ministry of Education, School of Pharmacy, Shihezi University, Shihezi 832003, China

**Keywords:** *Pseudomonas aeruginosa*, PqsR, virtual screening, pharmacophore modeling, molecular docking, molecular dynamics

## Abstract

*Pseudomonas aeruginosa* is a clinically challenging pathogen due to its high resistance to antibiotics. Quorum sensing inhibitors (QSIs) have been proposed as a promising strategy to overcome this resistance by interfering with the bacterial communication system. Among the potential targets of QSIs, PqsR is a key regulator of quorum sensing in *Pseudomonas aeruginosa*. However, the current research on PqsR inhibitors is limited by the lack of diversity in the chemical structures and the screening methods. Therefore, this study aims to develop a multidimensional screening model for PqsR inhibitors based on both ligand- and receptor-based approaches. First, a pharmacophore model was constructed from a training set of PqsR inhibitors to identify the essential features and spatial arrangement for the activity. Then, molecular docking and dynamics simulations were performed to explore the core interactions between PqsR inhibitors and their receptor. The results indicate that an effective PqsR inhibitor should possess two aromatic rings, one hydrogen bond acceptor, and two hydrophobic groups and should form strong interactions with the following four amino acid residues: TYR_258, ILE_236, LEU_208, and GLN_194. Moreover, the docking score and the binding free energy should be lower than −8 kcal/mol and −40 kcal/mol, respectively. Finally, the validity of the multidimensional screening model was confirmed by a test set of PqsR inhibitors, which showed a higher accuracy than the existing screening methods based on single characteristics. This multidimensional screening model would be a useful tool for the discovery and optimization of PqsR inhibitors in the future.

## 1. Introduction

*Pseudomonas aeruginosa* is a multifunctional Gram-negative bacterium [1]. It can cause inflammation, chronic persistent lung infection, and cystic fibrosis, and it has a high mortality rate [2]. *P. aeruginosa* exhibits strong drug resistance because it limits the penetration rate of antibiotic molecules into cells by producing biological enzymes, reducing the permeability of the outer membrane, and promoting the efflux of antibiotics, so that it can quickly adapt to drugs [3]. These resistance mechanisms are primarily regulated by cell density-dependent intercellular communication systems, also known as quorum sensing (QS).

QS is a chemical communication process used by bacteria to regulate their collective behavior [4]. *P. aeruginosa* controls its virulence and biofilm formation through QS. Biofilms are regulated by the secretion and sensing of a series of signaling molecules referred to as autoinducers [2]. Recent studies [5] have reported that, compared with traditional antibiotics, quorum sensing inhibitors (QSIs) exert less selective pressure on *P. aeruginosa* in terms of drug resistance; therefore, their own drug resistance evolution is also reduced [6]; this is highly effective against Gram-negative bacteria. Therefore, the current mainstream research and development direction for inhibitors of *P. aeruginosa* is to design inhibitors targeting QS systems.

Currently, there are several QSI studies on *P. aeruginosa*, primarily focusing on las, rhl, and pqs systems [7]. The key virulence regulatory receptor PqsR (also known as MvfR), an important target of the pq system, has recently attracted increasing attention from researchers. A series of studies on PqsR inhibitors have shown that PqsR inhibitors can effectively inhibit *P. aeruginosa* biofilm formation and reduce the expression of virulence factor genes [8,9].

However, although there have been some structure–activity relationship (SAR) studies on inhibitors targeting the parent core of PqsR, most of these studies only explored the SAR of a few compounds of the same parent core, inferred the SAR based on experimental data, or verified the experimental results using docking and molecular dynamics simulations. Relevant systematic reviews are also only summaries of the SAR conclusions, and a set of hierarchical virtual screening criteria for PqsR inhibitors has not been established to guide the synthesis and virtual screening of PqsR inhibitors.

We found that there are currently multi-target ligand-based studies [10,11] in other drug design aspects, and this method is feasible and effective. Therefore, this study aimed to develop a set of virtual screening criteria for PqsR inhibitors based on a systematic study of the SAR of PqsR inhibitors. First, we constructed a database of PqsR inhibitors using systematic reviews, including training and testing sets. A suitable pharmacophore model was constructed based on the ligand level to identify the active groups and spatial distribution of the PqsR inhibitors. At the ligand–receptor level, molecular docking, molecular dynamics simulations, and other techniques were used to discover the core indicators of the key interactions between PqsR inhibitors and their receptors. Then, a systematic SAR-based virtual screening model was constructed; that is, the first-level screening of active compounds was based on the ligand level, and the second-level screening of potential candidate compounds was based on the ligand–receptor interaction level. Finally, our test set was screened using the existing screening methods, and the higher accuracy and superiority of multi-level screening were reflected through horizontal comparison.

## 2. Results and Discussion

### 2.1. Database Results

We obtained a total screening of ten articles [2,12,13,14,15,16,17,18,19,20] on PqsR inhibitors in Table S1, and 283 compounds from these articles were built as a database in Table S2. The training set pharmacophore, shown in Table S3, consisted of the top 10% of the database’s activity (28 top-active compounds) and a decoy of 28 inactive compounds generated from the website https://dude.docking.org/ [21] (18 July 2023) in a 1:1 ratio. The molecular docking training set is shown in Table S4 and consists of 20 active and 20 non-active compounds in the database. The molecular dynamics simulation training set is shown in Table S5 and consists of five active compounds and five non-active compounds randomly selected from the database. The test set is shown in Table S6 and consists of 74 active compounds in the top 30% of the database with better activity and 1600 non-active bait compounds.

### 2.2. Pharmacophore Results of the Training Set and Its Validation

**Figure 1:**
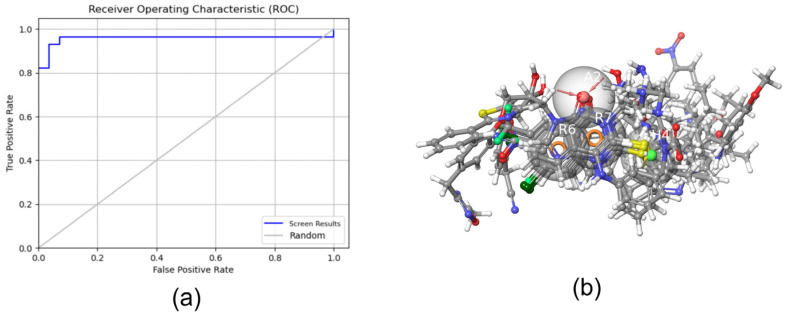
(**a**) ROC curves for AHRR_2, and (**b**) 3D structure of the pharmacophore training set matched to the AHRR_2 pharmacophore (The translucent sphere represents the pharmacophore group, the red sphere represents the acceptor feature, the orange rings represent the aromatic ring features, and the green sphere represents the hydrophobic region. Atoms are marked with different colors - gray for carbon atoms, blue for nitrogen atoms, light red for oxygen atoms, white for hydrogen atoms, and dark green for chlorine atoms).

Using the *Develop Pharmacophore Model* module to construct and validate the pharmacophore model (Table 1), we determined that the ROC of the pharmacophore model AHRR_2 was 0.96 and its AUC was 0.74, both >0.7 (Figure 1a). Therefore, it served as a candidate pharmacophore model. Further analysis of AHRR_2 showed that the model was composed of a hydrogen bond acceptor feature, two aromatic ring features, and one or two hydrophobic regions. This is also in accordance with the basic structure of quinazolinones and quinolones, which consist of an aromatic ring and a six-membered nitrogen heterocycle forming two aromatic groups. The carbonyl group in the heterocycle is a hydrogen-bond acceptor, whereas the long carbon chain is in the hydrophobic region. The active compounds in the pharmacophore training set were matched with the AHRR_2 pharmacophore (Table 2 and Figure 1b), and the good degree of matching indicated that the pharmacophore AHRR_2 can identify the key active groups and the spatial distribution of PqsR inhibitors. Therefore, we determined the following ligand-based screening criterion: the PqsR inhibitor should exactly match the four pharmacophore groups in AHRR_2. 

### 2.3. Molecular Docking Results of the Training Set

**Figure 2:**
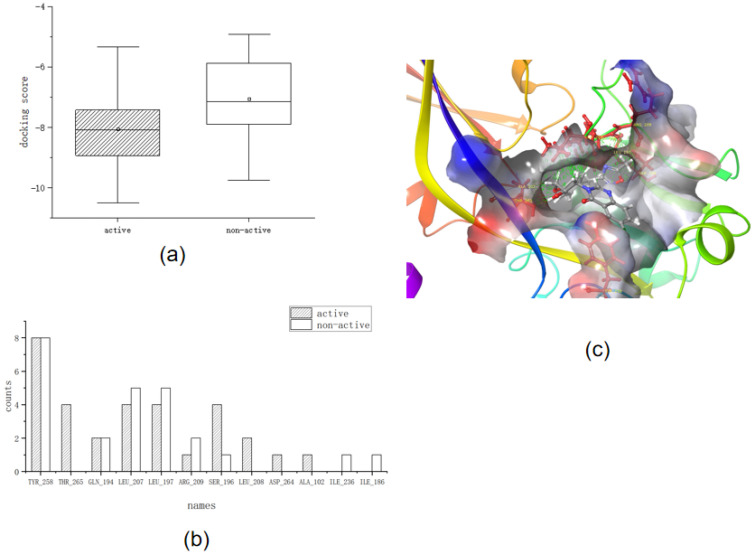
(**a**) Box plot of docking scores of the training set. (**b**) Frequency plot of key interactions of the training set. (**c**) Three-dimensional diagram of key interactions of the training set (Amino acid residues are marked in dark red, carbon atoms in gray, nitrogen atoms in blue, oxygen atoms in light red, and hydrogen atoms in white).

The *Ligand docking* module was used to construct and verify molecular docking criteria. Twenty active and twenty non-active compounds were analyzed in the training set. Table 3 and Figure 2a show the docking scores for the molecular docking of the training set, and Figure 2b,c show the key interactions of the training set.

First, we analyzed the docking scores. We determined that the main part of the docking score box plot ranged from −7.5 to −10 for active compounds, whereas the docking score box plot body ranged from −6 to −8 for non-active compounds. This indicated that the overall docking score of the active compounds was lower than that of the non-active compounds, which reflected a better interaction between the ligand and the receptor-docking conformation of the active compounds than that of the non-active compounds.

Second, we analyzed key interactions. Most of the active compounds interacted with TYR_258 through water bridges with the carbonyl group on the heterocycle. However, ARG_209, LEU_208, and others interact with hydrophobic groups such as hydrogen or methyl groups of long carbon chains. THR_265 is unique in that it reacts with long chains of nitrogen substituents close to the heterocyclic carbonyl group, such as the hydroxyl group of **16**. From the frequency analysis table of amino acid residues, we determined that TYR_258, LEU_197, LEU_207, SER_196, and THR_265 were frequently involved in this interaction. The higher the frequency of the interaction, the more likely it was to be a key amino acid residue for ligand binding.

Thus, we determined the screening criteria in molecular docking to be as follows:

1. The docking score should be <−8.
2. The structure of the compound requires two aromatic rings that are directly connected and a hydrophobic long chain structure such as a carbon chain or benzene ring, which is inserted into the protein pocket for anchoring (the pocket is preferably hydrophobic as a whole).
3. The compound should interact with the six amino acid residues TYR_258, LEU_197, LEU_207, SER_196, THR_265, and LEU_208.
4. The hydrophobic group in the structure of the compound is close to ARG_209 (2.26 − 6.91 Å) or the compound interacts with ARG_209.

### 2.4. Molecular Dynamics Simulations and Binding Free Energy Results of the Training Set

**Figure 3:**
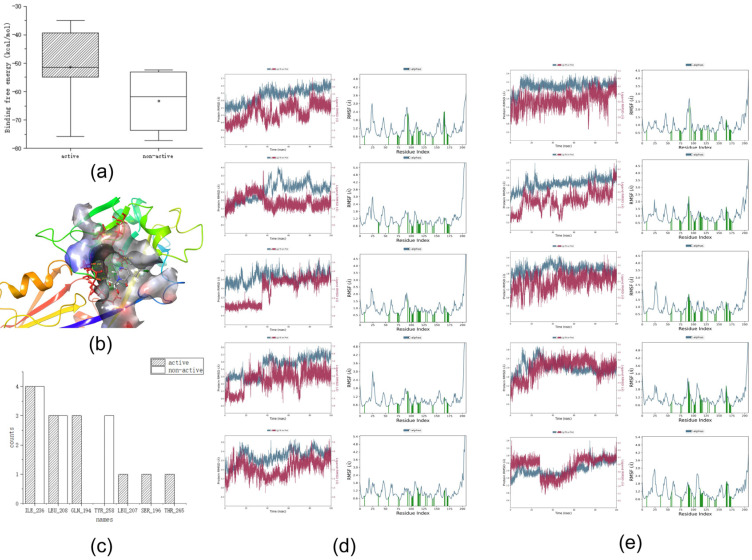
(**a**) Box plot of the training set binding free energy. (**b**) Frequency plot of key interactions of the training set dynamics (The colors in the figure indicate the same content as in Figure 2c). (**c**) Three-dimensional plot of key interactions of the more active compounds of the training set dynamics. (**d**,**e**) RMSD (The red line represents ligand RMSD and the blue line represents Cα RMSD. (Cα is the backbone carbon before the carbonyl atom in the amino acids)) and RMSF (The blue line represents Cα, and protein residues interacting with the ligand are labeled with green vertical bars.) of compounds with better and worse activity in the training set.

The *Molecular Dynamics* module in Desmond was used to perform molecular dynamics simulations, and the *MMGBSA* module was used to calculate the binding free energy. Five active and five non-active compounds were randomly selected for molecular dynamics simulation. Figure 3a shows the binding free energies of the training set, Figure 3b,c show the interactions in the dynamics, and Figure 3d,e show the RMSD and RMSF values in the dynamic simulation.

First, we analyzed the RMSF and RMSD. Compounds with five activities in the training set exhibited large fluctuations in the early stage. Analysis of the three-dimensional (3D) structure map showed that if the hydrophobic tail was a long carbon chain, it would lead to large fluctuations in the RMSF and RMSD values of the compounds. This may be due to the heterogeneous interactions between the carbon chain and the surrounding hydrophobic groups, such as those in compound 106. However, when a benzene ring or another nitrogen-containing heterocyclic ring is added to the hydrophobic end, such as in compound 82, the initial fluctuation is small and stability can be achieved quickly. This may be because the benzene ring or nitrogen-containing hydrophobic heterocyclic ring is relatively structurally stable, and it is difficult to change the spatial structure position because of the surrounding water molecular force field. Simultaneously, we observed that the RMSF and RMSD values of the compound tended to be stable at approximately 80 ns; therefore, we focused on the analysis of the key force magnitude after stabilization (80–100 ns).

Second, we analyzed the dynamic interactions. From the active compounds, it can be concluded that TYR_258, ILE_236, LEU_208, and GLN_194 interacted with all the active compounds over a long time. This phenomenon may indicate that TYR_258, ILE_236, LEU_208, and GLN_194 are the key amino acids that interact with the compounds during the molecular dynamics simulation.

Finally, the binding free energies were analyzed. We determined that the binding free energy box plots of active compounds ranged from −40 to −55 kcal/mol in the main part, whereas those of non-active compounds ranged from −53 to −70 kcal/mol (Figure 3). No significant differences were observed between the binding free energies of active and non-active sites. Because the binding free energy reflects the binding capacity, a lower value indicates that the compound is tightly bound to an amino acid residue. Therefore, considering the above analysis, we hypothesize that the binding free energy of an active compound should be <−40 kcal/mol.

After summarizing the above, we determined the following screening criteria for molecular dynamics:

1. The analysis of the binding free energy is best performed after stabilization (80–100 ns).
2. The compound should, if possible, interact continuously with the following four amino acid residues: TYR_258, ILE_236, LEU_208, and GLN_194.
3. The compound should, if possible, have a binding free energy (dG) of <−40 kcal/mol, and the main part should range from −40 to −55 kcal/mol.

### 2.5. Pharmacophore Results of the Test Set

**Figure 4:**
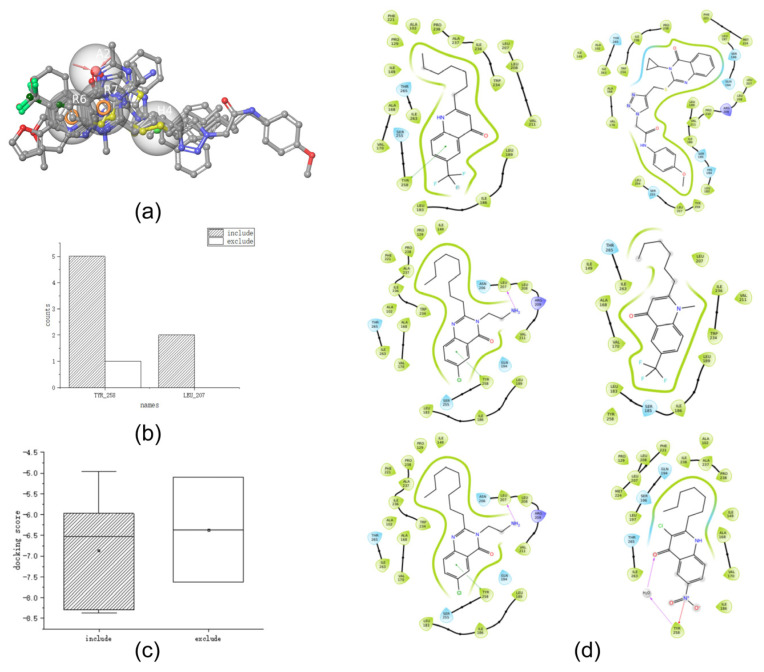
(**a**) Three-dimensional structure of the top ten pharmacophores of the test set matched with the AHRR_2 pharmacophore (The colors in the figure indicate the same content as in Figure 1b), (**b**) box diagram of test set docking scores, (**c**) frequency plot of key interactions for docking of the test set, and (**d**) 2D ligand interaction diagram of the test set (Green arrows represent π-π stacking. Purple arrows represent H-bonds).

After importing the test set into the pharmacophore, the top ten compounds were analyzed from high to low pharmacophore scores (Table 4). Following the analysis, all results were in line with the conclusions obtained from the training set; that is, all four pharmacophore groups in AHRR_2 were matched and the 3D structure diagram was constructed (Figure 4a), which is in line with the conclusions drawn in Section 2.2. Therefore, all ten compounds were retained for further verification.

### 2.6. Molecular Docking Results of the Test Set

Based on the pharmacophore results above, we proceeded with further validation using the docking scores and key interactions. Table 5 and Figure 4b,c show the docking scores and key docking interaction diagrams, and Figure 4d shows the 2D docking diagram of the active compounds of the test set with protein amino acid residues. Based on the docking score analysis, the docking scores of **232**, **56**, and **107** were all <−8 kcal/mol, which was consistent with the docking score conclusion drawn in Section 2.3. Key docking interaction analysis showed that compounds **232**, **56**, **107**, and **253** interacted with TYR_258. Furthermore, compounds **183** and **280** interacted with LEU_207, and the hydrophobic groups on these compounds were close to or interacted with ARG_209. These results are consistent with those obtained in Section 2.3 for key interactions between PqsR inhibitors and PqsR receptors. Together with the docking scores and docking interaction analyses described above, these six compounds fit the conclusions of molecular docking in the training set. Therefore, these six compounds were retained for further validation. Four other compounds were excluded because they either failed to interact with the key amino acid residues (TYR_258, LEU_197, LEU_207, SER_196, THR_265, and LEU_208) or had docking scores of >−8 kcal/mol.

### 2.7. Molecular Dynamics Simulations and Binding Free Energy Results of the Test Set

**Figure 5:**
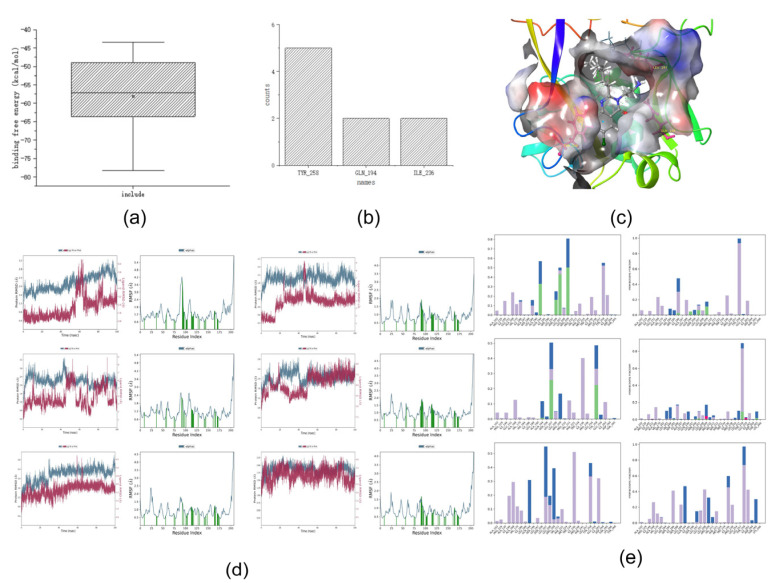
(**a**) Box plot of binding free energy in the dynamics of the test set, (**b**) frequency plot of key interactions of the dynamics of the test set, (**c**) 3D diagram of key interactions in molecular dynamics simulations of the test set (The colors in the figure indicate the same content as in Figure 2c), (**d**) RMSD and RMSF of the test set (The colors in the figure indicate the same content as in Figure 3d,e), and (**e**) contact_histogram (Green are H-bonds, purple are hydrophobic bonds, red are ionic bonds, blue are water bridges).

Based on the molecular docking results, further validation was performed from molecular dynamics simulations and binding free energies. Table 6 and Figure 5a show the binding free energy results of the test set, and Figure 5b,c show the persistent interactions between the compounds and amino acid residues in the molecular dynamics simulations. Figure 5d shows the RMSD and RMSF values. Figure 5e shows the long-term interaction between compounds and amino acid residues.

First, we analyzed the RMSD and RMSF. The values of these six compounds tended to be stable at 80 ns and 100 ns, which is in line with the conclusion of Section 2.4. Second, we analyzed dynamic interactions, as shown in Table 6 (note: when the interaction fraction between the amino acid residues and the compound is >0.5, it is considered to have a long-term interaction, indicated by “√”). We determined that six compounds interacted with three amino acid residues, TYR_258, GLN_194, and ILE_236, for a long time. For example, compounds 280 and 56 had a persistent interaction with GLN_194, and 183 had a persistent interaction with ILE_236, which is consistent with the conclusions of Section 2.4. Finally, we analyzed the binding free energy and determined that the binding free energy dG of the above six compounds was <−40 kcal/mol, which was in line with the conclusion of Section 2.4 regarding the active binding free energy. Collectively, the results of the molecular dynamics simulations and binding free energies suggested that all six of the above candidates were active compounds.

In this study, a multidimensional virtual screening model for PqsR inhibitors was constructed using the training set, and multidimensional hierarchical screening was performed on the test set based on the evaluation criteria of the virtual screening model, including pharmacophore screening based on ligand conformation. Based on the screening of molecular docking of receptor–ligand interactions, molecular dynamics simulations, and binding free energy, six candidate compounds were selected. These six compounds were all active, indicating that the multidimensional virtual screening model constructed in this study can reliably identify active PqsR inhibitors.

### 2.8. Comparison of Results with Existing Screening Criteria

To further demonstrate the superiority of the multidimensional screening model, we selected two articles that searched for PqsR quorum sensing inhibitors using computer software [20,21]. The difference is that these two studies only considered a single-dimension aspect, that is, molecular docking and molecular dynamics simulations of compounds and binding free energy calculations based on receptors only. Among them, Vieira [22] et al. suggested that the two residues Ile_186 and Tyr_258 may be the key to the activation mechanism of MvfR (PqsR). Shandil [20] et al. suggested that the higher level of pqs inhibition may be due to the hydrogen bond interaction with GLN_194, LEU_207, THR_265, and SER_196; the pi sulfur bond with PHE_221; and the hydrophobic interaction with TYR_258 and LEU_197. Therefore, we used the above conclusions as screening criteria and verified them using the test set in this study.

First, we downloaded two protein crystal structures (PDBID: 4JVI and 6B8A) selected from the two studies, preprocessed the molecules in the test set, and performed molecular docking with the two crystal structures. The compounds were ranked from high to low based on the absolute value of the docking scores, and the compounds that were consistent with the conclusions in the literature were retained. The top ten compounds were analyzed and the screening accuracy was calculated (Table 7).

The results showed that only seven of the top ten compounds were active after protein structure 4JVI docking, including three false-positive results, with an accuracy of 57.1%. After docking the protein structure 6B8A, the hit rates of the top ten compounds were not high enough (55.6% and 71.4%, respectively) (Table 7). In contrast, the ligand-based pharmacophores followed by receptor-based docking screening used in this study identified six active compounds out of the top ten docking scores, and no false positives were indicated. This reflects the advantages of multidimensional screening.

## 3. Discussion

In this study, a database was constructed based on existing studies on the reported inhibitors of PqsR. Based on this database, a pharmacophore model of the PqsR inhibitor was established, and key interactions between the PqsR inhibitor and its receptor were investigated using docking and molecular dynamics techniques. Finally, a set of multidimensional virtual screening criteria considering both the structural characteristics of the ligand and the key interaction characteristics of the receptor and ligand was successfully developed.

As an important aspect of the pq system, the role of PqsR should not be underestimated. Once HHQ/PQS activates PqsR, it induces its own biosynthesis with an exponential increase in the concentration of signaling molecules [23]. Some studies have shown that PqsR can also bind to and directly control various sites expressed throughout the genome of *P. aeruginosa*, including major regulators such as LasR and RhlR, as well as genes involved in protein translation, secretion, and response to oxidative stress [8]. Therefore, it is important to identify PqsR inhibitors as a potential strategy to reverse *P. aeruginosa* resistance. However, currently, the discovery and screening strategies for inhibitors are primarily traditional structure derivation and activity screening, which are of great application value to accelerate the discovery of inhibitors.

Virtual screening is an effective method to identify candidate compounds; however, the use of a single technology (single ligand-based or single key interaction-based) often leads to an increase in false positives or false negatives. For example, Ji et al. [24] used 5′ -O-(N-acylsulfonyl) adenosine (acyl-AMS) and related compounds to mimic homologous and tightly bound acyl-AMP intermediates to inhibit such enzymes by designing inhibitors related to the target PqsA upstream of the PQS system. This operation has its own rationality, but it cannot control the activity of the newly designed compound because of the lack of structure–activity relationship analysis. “Vinyl sulfonamide analogues 6 and 7 were excluded due to a lack of biochemical potency”, the article states. In contrast, salicyl-AMS (3), salicyl-AMSN (4), and benzoyl-AMS are inhibitors of quinolone production but are much less potent [24]. To further improve the accuracy of virtual screening, systematic integration of pharmacophore modeling, molecular docking, molecular dynamics, and other methods is required to construct multidimensional virtual screening criteria. For example, Luo et al. [25] applied Pharmacome modeling, virtual screening, molecular docking, ADMET, and molecular dynamics simulations to identify potential PD-L1 inhibitors in a marine natural product library. In this study, pharmacophore-based virtual screening was performed using a marine natural product library of 52765 compounds according to the generated pharmacophore model. Twelve compounds conforming to all the pharmacophore properties were generated, and molecular docking was performed. Finally, the selected compounds, 51320 and 37080, were analyzed using molecular dynamics simulations, which confirmed that the compound and protein binding sites were stable. In addition, high dG binding values were observed for MM/GBSA, as calculated from individual trajectories, indicating the long-term stability of the selected protein–ligand complexes. Compound 51320 exhibited low toxicity and good activity. The data and results obtained also reflect the feasibility and accuracy of virtual screening based on various SAR analysis techniques.

At present, some studies on the structure–activity relationship and virtual screening of PqsR inhibitors exist. For example, Zender et al. [26] further synthesized compounds based on 2-aminoxediazole as the backbone, evaluated their activity in Escherichia coli, and analyzed their structure–activity relationship. Based on the aminopyridine derivatives of fragments, Zender et al. [27] determined the activity of the fragment compounds and screened new inhibitors with aminopyridine as the backbone using the fragment growth method. Vieira et al. [22] performed molecular docking, molecular dynamics simulation, and MM/GBSA free energy calculations on 64 known drugs and protein pockets to confirm docking prediction and clarify interaction modes; the ROC and AUC were measured to be approximately 0.5. The last five compounds were identified as promising PqsR inhibitors. The above studies fully prove the feasibility of traditional compound screening based on structure–activity relationships, fragment growth, and computer-assisted virtual screening for drug development; however, they are only based on a single dimension of screening: whether it is an activity assay based on a certain skeleton or a simple virtual screening based on receptors, certain limitations exist. It is reasonable to consider that if the evaluation index of virtual screening considers both the structural characteristics of the ligand and the key ligand–receptor interactions, the prediction accuracy will be improved.

Therefore, in this study, we used a ligand-based pharmacophore model, receptor–ligand molecular docking, and molecular dynamics simulation techniques to analyze the structure–activity relationships of compounds to construct a multidimensional virtual screening model and standard for PqsR inhibitors. The first level of ligand-based pharmacophore screening concluded that compounds should cover two aromatic rings, a hydrogen bond acceptor, and two hydrophobic groups; The second level of screening based on molecular docking and molecular dynamics simulation of the receptor concluded that the docking score should be <−8 kcal/mol, the compound should interact continuously with four amino acid residues as much as possible (TYR_258, ILE_236, LEU_208, and GLN_194), and the dG of the compound should be <−40 kcal/mol. This test set was used for further horizontal verification. By comparing the results of the test set screened according to the screening criteria of a single dimension with the results of multidimensional screening in this study, we determined that the accuracy of multidimensional screening was higher. Therefore, we believe that this virtual screening model can be applied for the subsequent development of PqsR inhibitors for *P. aeruginosa*.

However, the results of the present study require further improvement. First, the sample size was insufficiently large. Only ten studies were included in this review, and the total number of compounds in these studies was not more than 300. Further expansion of the number of compounds is required. Second, the methods used to measure the activity data and the strains used were different in different studies, and the activity indices were not the same. Some studies have used IC50 values, whereas others have used MIC values. Therefore, at present, our evaluation model primarily focuses on qualitative indicators, that is, whether the candidate compounds are PqsR inhibitors, and cannot make further quantitative judgments. Despite the limitations of this study, our research group is committed to studying drug-resistant bacteria. Based on the multidimensional virtual screening model of PqsR inhibitors constructed in this study, we will continue to perform the strategy of “virtual screening-activity validation-optimization model” to find new PqsR inhibitors. In this process, the database of PqsR inhibitors was continuously expanded and the evaluation criteria for pharmacophores, molecular docking, and molecular dynamics were optimized to continuously improve the prediction accuracy of the multidimensional virtual screening model of PqsR inhibitors.

## 4. Materials and Methods

### 4.1. Establishment of Database

To construct the virtual screening criteria, the two keywords PqsR inhibitor AND structure–activity relationship, PqsR AND inhibitor were searched in Web of Science and PubMed. Articles that met the following criteria were included: 1. PqsR inhibitors against *P. aeruginosa*; 2. Inhibitors included in the articles were quinazolinone and quinolone compounds obtained by the modification of the HHQ skeleton, and a circulating amount of PqsR’s natural ligands 2-heptyl-4-quinolone; 3. In vitro experiments were performed, and the data on the inhibition rates are available (concentration of drug responsible for 50% of maximal effect [IC50] and minimal inhibitory concentration [MIC]).

We selected the top 10% of active compounds in the database to construct the qualitative pharmacophore model, that is, the training set of the pharmacophore. The top 20 compounds with the best activity in the database were defined as active compounds, and the bottom 20 compounds with the worst activity were defined as non-active, as the training set for molecular docking. Then, we manually selected 5 active and 5 non-active compounds based on the conformation similarity of natural ligands and docking score (top 50%) as the training set of the molecular dynamics simulations. Simultaneously, 30% of the active compounds and 1600 decoys were selected as the test set (decoys were automatically generated from the top 10% of active compounds using the website https://dude.docking.org/ [21]).

### 4.2. Pharmacophore Model Construction

Develop Pharmacophore Model developed by Maestro was used to construct this model [28]. The pharmacophore was based on the training set, and the specific parameters were as follows: at least 25% of the groups of the compound were matched in Hypothesis Settings, the number of features in the hypothesis was set to 4–7, the preferred minimum number of features was set to 5, and the hypothesis difference criterion was set to 1.

The ROC curve [29] reflects the relationship between sensitivity and specificity. The area under the ROC curve (AUC) was determined. The higher the AUC value, the closer the curve is to the upper-left corner, and the greater the AUC, the higher the prediction accuracy. Therefore, the pharmacophores included in this study satisfied ROC > 0.9 and AUC > 0.7.

### 4.3. Molecular Docking

To perform molecular docking, we used the following modules in Ligand Docking: RCSB protein database (http://www.rcsb.org/ [30]) was used to download X-ray crystal structures (PDB ID: 6YIZ, resolution 2.16 Å [31]). Protein Preparation Workflow [32] developed by Maestro was used for receptor protein preparation. LigPrep [33] was used to minimize the energy of small molecules, Receptor Grid Generation was used to generate docking pockets, and Glide XP [34] was used to explore the interaction between PqsR inhibitors and their receptors (the scaling factor was set to 0.86, and the rest were set as default). The docking score indicates the docking interaction between the ligand and receptor. Generally, the lower the docking score and the lower the energy, the better the interaction between the ligand and the receptor docking conformation (note: it cannot directly indicate the actual activity of the receptor–ligand, for reference only). Ligand interactions were used to visualize the two-dimensional (2D) docking results, and amino acid residues with notable key roles were shown [35]. In this study, rigid docking was adopted for rapid matching between compounds and receptors. Then, molecular dynamics simulations were applied to further analyze the changes in conformation as well as key interactions.

In the present study, the docking scores were ranked from high to low to divide the range in which the activity docking scores were located. The differences in the 2D docking results between active and non-active compounds in the training set were used to identify amino acid residues closely related to the activity of PqsR inhibitors. For the test set, the docking score ranges of the active compounds obtained from the training set were screened for important amino acid residues to obtain candidate active compounds.

### 4.4. Molecular Dynamics Simulation

For the molecular dynamics simulation, we used the following modules: System Builder in desmond [36] to build the water molecular environment, Minimization to minimize the overall docking energy, Molecular Dynamics to perform the molecular dynamics simulation, and MMGBSA [32] in the Prime module was used to calculate the polar solvation energy and binding free energy through the generalized Born model. The script defaults to build an implicit solvent model, and The Simulation Interaction Diagram Module was used to view the molecular dynamics simulation results. In addition, to better distinguish the compounds with good and poor activity, we applied the following settings: in the docking, the simulation time was set to 100 ns; the approximate number of frames was set to 10,000; the combined free energy was calculated when the overall energy was stable; we set the interval from the 8000th to the 10,000th frame, that is, 80–100 ns, and recorded data every ten frames. The temperature of the molecular dynamics simulation and the calculation of the binding free energy is 300 k, and the pressure is 1.01325 bar. The ensemble class is NPT—constant particle number (N), pressure (P), and temperature (T). This class is an isothermal–isobaric ensemble, which is the common experimental condition.

The Root Mean Square Deviation (RMSD) and Root Mean Square Fluctuation (RMSF) are metrics commonly used in structural biology and molecular dynamics simulations. They are used to measure the degree of difference between two structures as well as the vibration or fluctuation of atoms in a protein. In addition, we analyzed the Gibbs free energy. Binding free energy is an important concept in thermodynamics, which is a function of entropy and enthalpy changes and is used to predict orientation and equilibrium conditions during the running of molecular dynamics simulations.

We identified the key interactions of PqsR inhibitors, binding free energies, and evaluation metrics such as RMSD and RMSF in the training set. Based on the above criteria, candidate compounds obtained from the pharmacophore and molecular docking screening test sets were evaluated to determine the final screening results.

## Figures and Tables

**Table 1:** Pharmacophore training set and matching AHRR_2 pharmacophore table.

Hypothesis	Phase Hypo Score	ROC	AUC	Matches	Total Actives	Ranked Actives
AHRR_2	1.07	0.96	0.74	4 of 4	28	27
AHHR_3	0.96	0.64	0.66	4 of 4	28	18
AHHR_2	1.00	0.61	0.65	4 of 4	28	17
AHHR_5	0.95	0.60	0.64	4 of 4	28	17
HHRR_1	0.96	0.54	0.63	4 of 4	28	15
AHRR_3	1.04	0.53	0.63	4 of 4	28	15
AHRR_1	1.16	0.53	0.63	4 of 4	28	15
AHHR_1	1.01	0.50	0.62	4 of 4	28	14
AHHR_4	0.98	0.45	0.60	4 of 4	28	13
HHRRR_2	0.58	0.45	0.58	4 of 5	28	14
HHRR_2	0.95	0.43	0.60	4 of 4	28	12
HHRRR_1	0.61	0.38	0.56	4 of 5	28	12

**Table 2:** Pharmacophore training set and matching AHRR_2 pharmacophore table.

No.	Code	Matched Ligand Sites	No.	Code	Matched Ligand Sites	No.	Code	Matched Ligand Sites	No.	Code	Matched Ligand Sites
1	16	4	8	87	4	15	163	4	22	221	4
2	26	4	9	106	4	16	164	4	23	222	4
3	57	4	10	109	4	17	165	4	24	243	4
4	58	4	11	120	4	18	167	4	25	247	4
5	59	4	12	126	4	19	185	4	26	267	4
6	67	4	13	133	4	20	188	4	27	280	4
7	82	4	14	140	4	21	219	4	28	283	4

**Table 3:** Docking score table of the training set.

Activity			Non-Activity		
No.	Code	Docking Score	No.	Code	Docking Score
1	82	−10.495	21	76	−9.740
2	87	−9.763	22	141	−9.192
3	283	−9.660	23	79	−8.899
4	106	−9.285	24	145	−8.646
5	133	−8.967	25	31	−7.905
6	221	−8.905	26	231	−7.874
7	222	−8.697	27	274	−7.801
8	58	−8.446	28	111	−7.404
9	164	−8.249	29	6	−7.293
10	16	−8.131	30	96	−7.151
11	57	−8.041	31	276	−7.146
12	163	−7.923	32	49	−6.786
13	186	−7.851	33	44	−6.406
14	26	−7.728	34	197	−6.104
15	247	−7.661	35	257	−5.894
16	181	−7.201	36	147	−5.879
17	120	−6.871	37	256	−5.638
18	267	−6.016	38	200	−5.514
19	280	−5.776	39	151	−5.227
20	109	−5.339	40	217	−4.921

**Table 4:** Top ten compounds with pharmacophore fractions in the test set.

Rank	Compound	Matched Ligand Sites
1	253	4
2	56	4
3	232	4
4	5	4
5	107	4
6	280	4
7	C01833070	4
8	148	4
9	C00658248	4
10	183	4

**Table 5:** Docking results between test sets and proteins.

No.	Code	Docking Score	Conclusion 1	Conclusion 2	Conclusion 3	Retained
1	232	−8.369	√ *	√	√	yes
2	56	−8.369	√	√	√	yes
3	107	−8.223	√	√		yes
4	C00658248	−7.627		√		no
5	183	−6.790	√	√		yes
6	280	−6.261	√		√	yes
7	5	−6.170	√			no
8	253	−5.777	√	√		yes
9	C01833070	−5.159				no
10	148	−4.967	√			no

* “√” indicate that the compounds match the conclusions.

**Table 6:** Molecular dynamics simulation results of the test set and binding free energy of the test set.

No.	Code	Key Interactions				dG (kcal/mol)
		TYR_258	ILE_236	LEU_208	GLN_194	
1	280	√ *	√		√	−78.244 ± 4.28
2	232	√				−63.616 ± 4.43
3	183		√			−58.546 ± 5.42
4	253	√				−55.916 ± 4.55
5	56	√			√	−48.963 ± 4.73
6	107	√				−43.497 ± 4.39

* “√” indicate that the compounds match the conclusions.

**Table 7:** Multidimensional versus single-dimensional test set screening results.

Criteria	Article 1 ^1^		Article 2 ^2^	Our Study
Receptors	4JVI	6B8A	6B8A	6YIZ
Hit compounds	C08400469	C41350371	C41350371	232
	124	126	C35288781	56
	140	140	126	107
	126	120	140	183
	C96360389	134	C04834659	280
	133	C20906765	120	283
	C09116355	82	134	
			C20906765	
			82	
Accuracy rating	57.10%	71.40%	55.60%	100%

^[1]^ Vieira T F, Magalhães R P, Simões M et al. Drug Repurposing Targeting *Pseudomonas aeruginosa* MvfR Using Docking, Virtual Screening, Molecular Dynamics, and Free-Energy Calculations[J]. Antibiotics, 2022, 11(2): 185 [22]. ^[2]^ Shandil S, Yu T T, Sabir S et al. Synthesis of Novel Quinazolinone Analogues for Quorum Sensing Inhibition[J]. Antibiotics, 2023, 12(7): 1227 [20].

## Data Availability

Data presented in this study are available on request from the corresponding author.

## Supplementary Materials

The following supporting information can be downloaded at: https://www.mdpi.com/article/10.3390/ijms25031869/s1.
