# Supplementary material for: Multidimensional Criteria for Virtual Screening of PqsR Inhibitors Based on Pharmacophore, Docking, and Molecular Dynamics
Source: Int J Mol Sci. 2024 Feb 3;25(3):1869. doi: 10.3390/ijms25031869 (PMC10856439; doi:10.3390/ijms25031869)
Supplement: Supplementary file 1 [file ijms-25-01869-s001.zip › ijms-2797586-supplementary.pdf]

Table S1. Correspondence map of the literature

No.	Name
1	Optimization of anti-virulence PqsR antagonists regarding aqueous solubility and biological properties resulting in new insights in structure–activity relationships
2	A Pseudomonas aeruginosa PQS quorum-sensing system inhibitor with anti-staphylococcal activity sensitizes polymicrobial biofilms to tobramycin
3	Design and Evaluation of New Quinazolin-4(3H)-one Derived PqsR Antagonists as Quorum Sensing Quenchers in Pseudomonas aeruginosa
4	Discovery of Antagonists of PqsR, a Key Player in 2-Alkyl-4-quinolone-Dependent Quorum Sensing in Pseudomonas aeruginosa
5	In Silico and in Vitro-Guided Identification of Inhibitors of Alkylquinolone-Dependent Quorum Sensing in Pseudomonas aeruginosa
6	Novel quinazolinone disulfide analogues as pqs quorum sensing inhibitors against Pseudomonas aeruginosa
7	Novel quinazolinone inhibitors of the Pseudomonas aeruginosa quorum sensing transcriptional regulator PqsR
8	Structural Basis for Native Agonist and Synthetic Inhibitor Recognition by the Pseudomonas aeruginosa Quorum Sensing Regulator PqsR (MvfR)
9	Structure–functionality relationship and pharmacological profiles of Pseudomonas
10	aeruginosa alkylquinolone quorum sensing modulatorsSynthesis of Novel Quinazolinone Analogues for Quorum Sensing Inhibition

Table S2. All compounds in the database:

Code.	Structure	From	Code	Structure	From	Code	Structure	From
1	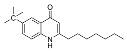	1	95	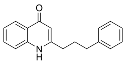	4	189	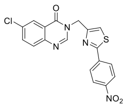	7
2	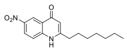	1	96	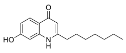	4	190	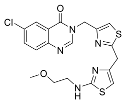	7
3	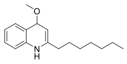	1	97	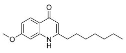	4	191	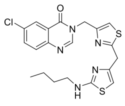	7
4	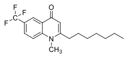	1	98	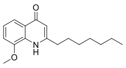	4	192	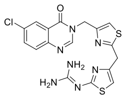	7
5	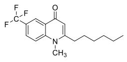	1	99	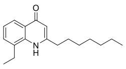	4	193	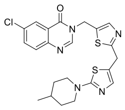	7
6	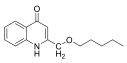	1	100	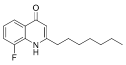	4	194	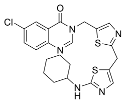	7

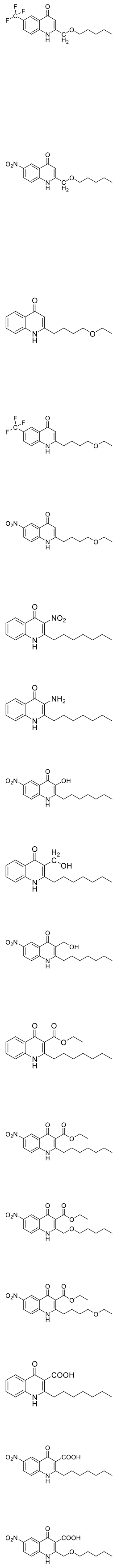

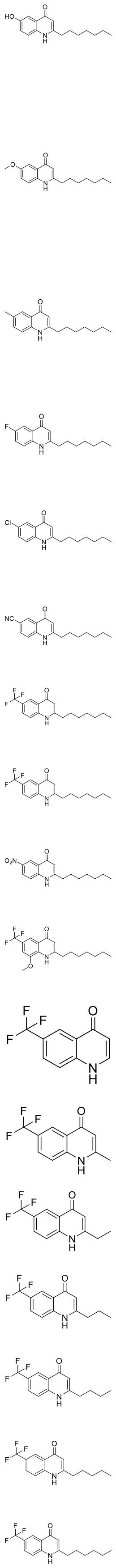

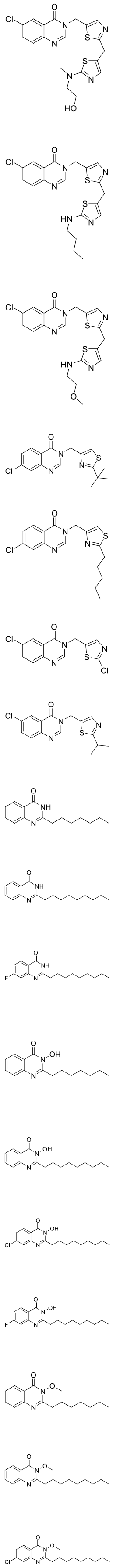

7	1	101	4	195	7
8	1	102	4	196	7
9	1	103	4	197	7
10	1	104	4	198	7
11	1	105	4	199	7
12	1	106	4	200	7
13	1	107	4	201	7
14	1	108	4	202	8
15	1	109	4	203	8
16	1	110	4	204	8
17	1	111	4	205	8
18	1	112	4	206	8
19	1	113	4	207	8
20	1	114	4	208	8
21	1	115	4	209	8
22	1	116	4	210	8
23	1	117	4	211	8

24	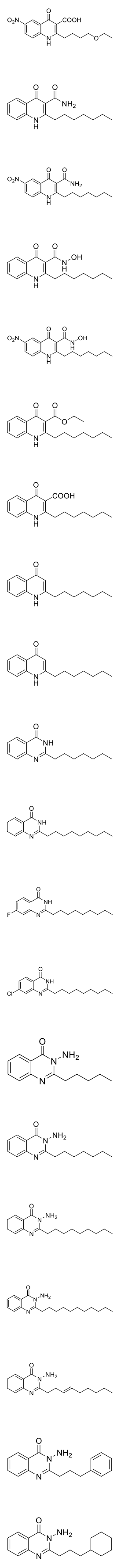	1	118	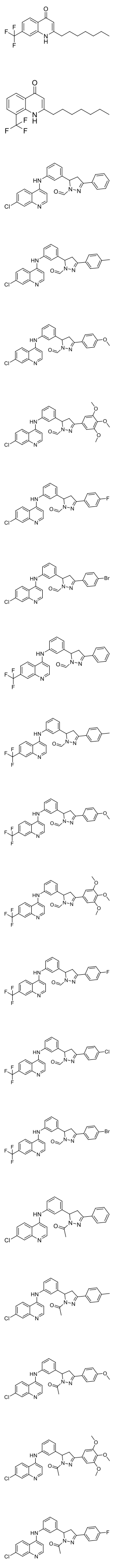	4	212	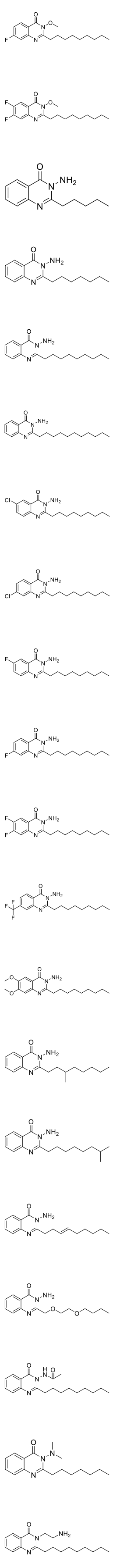	8
25		1	119		4	213		8
26		1	120		5	214		8
27		1	121		5	215		8
28		1	122		5	216		8
29		1	123		5	217		8
30		1	124		5	218		8
31		1	125		5	219		8
32		1	126		5	220		8
33		2	127		5	221		8
34		2	128		5	222		8
35		2	129		5	223		8
36		2	130		5	224		8
37		2	131		5	225		8
38		2	132		5	226		8
39		2	133		5	227		8
40		2	134		5	228		8
41		2	135		5	229		8
42		2	136		5	230		8
43		2	137		5	231		8

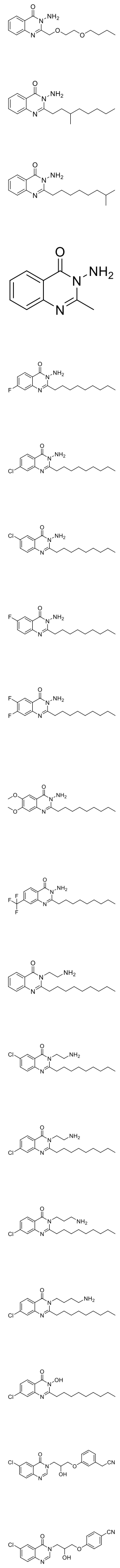

44	2
45	2
46	2
47	2
48	2
49	2
50	2
51	2
52	2
53	2
54	2
55	2
56	2
57	2
58	2
59	2
60	2
61	3
62	3

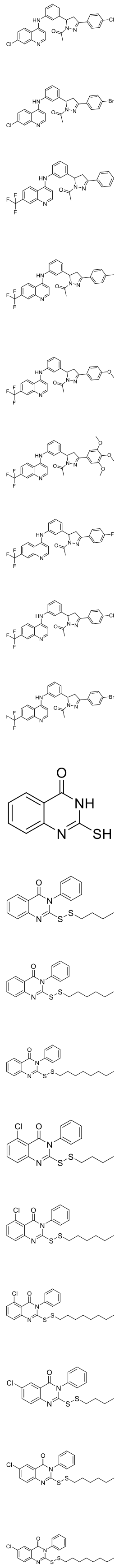

138	5
139	5
140	5
141	5
142	5
143	5
144	5
145	5
146	5
147	6
148	6
149	6
150	6
151	6
152	6
153	6
154	6
155	6
156	6

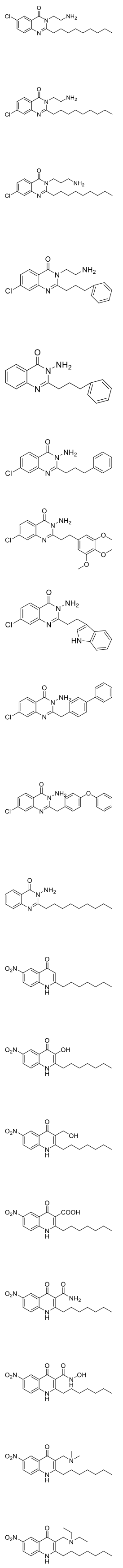

232	8
233	8
234	8
235	8
236	8
237	8
238	8
239	8
240	8
241	8
242	8
243	9
244	9
245	9
246	9
247	9
248	9
249	9
250	9

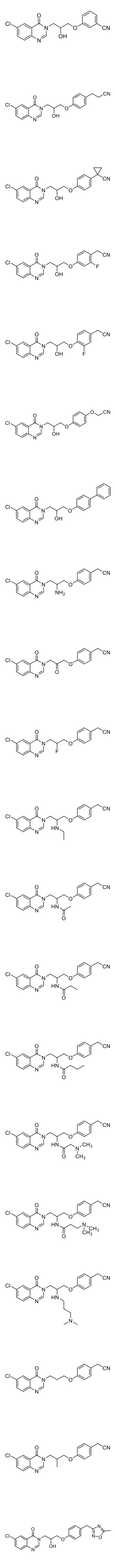

63		3
64		3
65		3
66		3
67		3
68		3
69		3
70		3
71		3
72		3
73		3
74		3
75		3
76		3
77		3
78		3
79		3
80		3
81		3
82		3

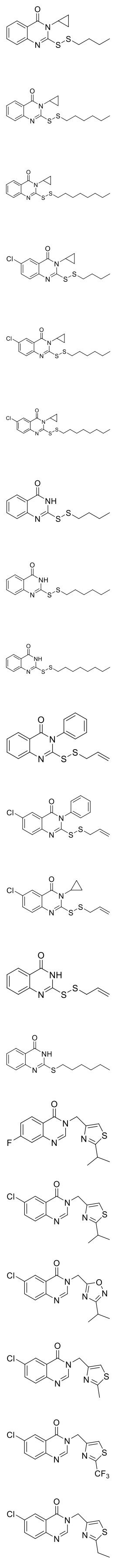

157		6
158		6
159		6
160		6
161		6
162		6
163		6
164		6
165		6
166		6
167		6
168		6
169		6
170		6
171		7
172		7
173		7
174		7
175		7
176		7

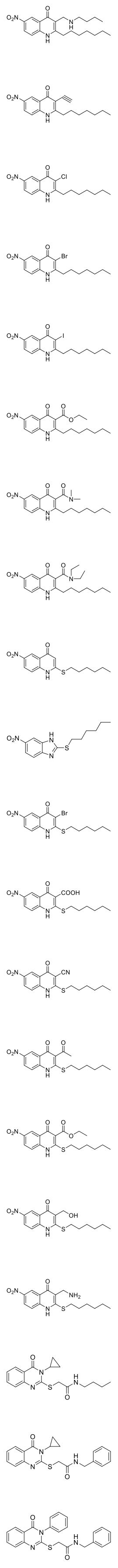

251		9
252		9
253		9
254		9
255		9
256		9
257		9
258		9
259		9
260		9
261		9
262		9
263		9
264		9
265		9
266		9
267		9
268		10
269		10
270		10

83	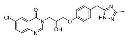	3	177	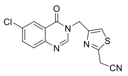	7	271	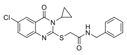	10
84	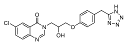	3	178	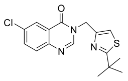	7	272	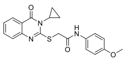	10
85	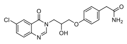	3	179	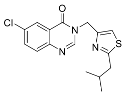	7	273	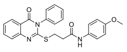	10
86	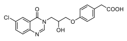	3	180	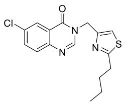	7	274	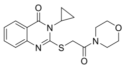	10
87	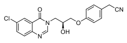	3	181	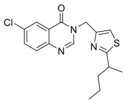	7	275	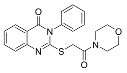	10
88	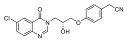	3	182	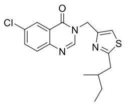	7	276	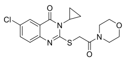	10
89	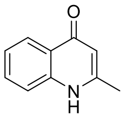	4	183	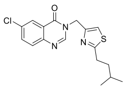	7	277	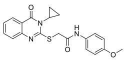	10
90	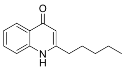	4	184	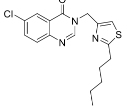	7	278	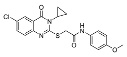	10
91	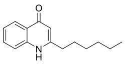	4	185	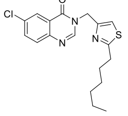	7	279	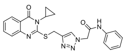	10
92	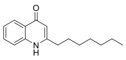	4	186	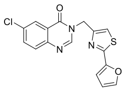	7	280	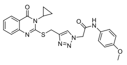	10
93	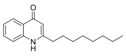	4	187	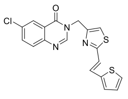	7	281	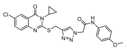	10
94	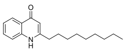	4	188	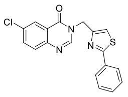	7	282	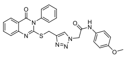	10
						283	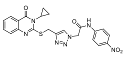	10

Table S3. Pharmacophore training set

Activity											
No.	Code	Reference	No.	Code	Reference	No.	Code	Reference	No.	Code	Reference
1	**16**	1	8	**87**	3	15	**163**	6	22	**221**	8
2	**26**	1	9	**106**	4	16	**164**	6	23	**222**	8
3	**57**	2	10	**109**	4	17	**165**	6	24	**243**	9

4	**58**	2	11	**120**	5	18	**167**	6	25	**247**	9
5	**59**	2	12	**126**	5	19	**185**	7	26	**267**	9
6	**67**	3	13	**133**	5	20	**188**	7	27	**280**	10
7	**82**	3	14	**140**	5	21	**219**	8	28	**283**	10
Decoyset (28[a],www.dude.docking.org[b],Cxxxxxxxx[c])											

a. The total number of decoyset

b. The website for generating decoyset

c. The code of decoyset

Table S4. Molecular docking for the training set

Activity						Non-activity					
No.	Code	Reference	No.	Code	Reference	No.	Code	Reference	No.	Code	Reference
1	**16**	1	11	**163**	6	21	**6**	1	31	**147**	6
2	**26**	1	12	**164**	6	22	**31**	1	32	**151**	6
3	**57**	2	13	**186**	7	23	**44**	2	33	**197**	7
4	**58**	2	14	**181**	7	24	**49**	2	34	**200**	7
5	**82**	3	15	**221**	8	25	**76**	3	35	**217**	8
6	**87**	3	16	**222**	8	26	**79**	3	36	**231**	8
7	**106**	4	17	**247**	9	27	**96**	4	37	**256**	9
8	**109**	4	18	**267**	9	28	**111**	4	38	**257**	9
9	**120**	5	19	**280**	10	29	**141**	5	39	**274**	10
10	**133**	5	20	**283**	10	30	**145**	5	40	**276**	10

Table S5. Molecular dynamics simulation for the training set

Activity			Non-activity		
No.	Code	From	No.	Code	From
1	**82**	3	6	**141**	5
2	**87**	3	7	**79**	3
3	**106**	4	8	**145**	5
4	**164**	6	9	**31**	1
5	**16**	1	10	**231**	8

Table S6. Test set

Activity					
No.	Code	From	No.	Code	From
1	**5**	1	38	**158**	6
2	**7**	1	39	**163**	6
3	**8**	1	40	**164**	6
4	**16**	1	41	**165**	6
5	**26**	1	42	**168**	6

6	**1**	1	43	**172**	7
7	**2**	1	44	**178**	7
8	**4**	1	45	**179**	7
9	**56**	2	46	**181**	7
10	**57**	2	47	**183**	7
11	**58**	2	48	**184**	7
12	**59**	2	49	**185**	7
13	**60**	2	50	**186**	7
14	**55**	2	51	**188**	7
15	**66**	3	52	**191**	7
16	**67**	3	53	**232**	8
17	**70**	3	54	**233**	8
18	**71**	3	55	**234**	8
19	**82**	3	56	**235**	8
20	**87**	3	57	**237**	8
21	**88**	3	58	**215**	8
22	**64**	3	59	**216**	8
23	**107**	4	60	**217**	8
24	**108**	4	61	**219**	8
25	**109**	4	62	**221**	8
26	**115**	4	63	**222**	8
27	**116**	4	64	**231**	8
28	**117**	4	65	**253**	9
29	**106**	4	66	**254**	9
30	**120**	5	67	**255**	9
31	**124**	5	68	**267**	9
32	**126**	5	69	**243**	9
33	**133**	5	70	**245**	9
34	**134**	5	71	**247**	9
35	**140**	5	72	**280**	10
36	**148**	6	73	**283**	10
37	**157**	6	74	**268**	10
Decoyset (1600[a],www.dude.docking.org[b],Cxxxxxxxx[c])					

a. The total number of decoyset

b. The website for generating decoyset

c. The code of decoyset
